# Influence of an Automatic Enrichment Device on Laying Hen Behavior and Plumage Condition

**DOI:** 10.3390/ani13060989

**Published:** 2023-03-08

**Authors:** Anna Riedel, Meryem Canci, Birgit Spindler, Nicole Kemper

**Affiliations:** 1 Institute for Animal Hygiene, Animal Welfare and Farm Animal Behaviour, University of Veterinary Medicine Hannover, Foundation, Bischhofsholer Damm 15, D-30173 Hannover, Germany; 2 WING (Science and Innovation for Sustainable Poultry Production), University of Veterinary Medicine Hannover, Foundation, Heinestraße 1, D-49377 Vechta, Germany

**Keywords:** laying hen, automatic enrichment, plumage condition, winter garden, behavioral observation

## Abstract

**Simple Summary:**

Environmental enrichment is widely seen as an improvement to animal welfare, and in modern laying hen husbandry a variety of enrichment materials are used. In this study, which was conducted on a German organic laying hen farm, an automatic enrichment device that doses grains on rough-coated plates was tested in different quantities. The animals’ behavior in the area around the device was observed via video recordings. Furthermore, the hens were scored for plumage damage during regular visits to check whether the enrichment device helped the hens not to develop feather pecking and cannibalistic behavior. The hens in general interacted less with their environment at the end of their husbandry period, but the offer of the enrichment device did not influence their preferences for performed behaviors. However, it was observed that hens offered a high number of enrichment devices stayed in the surrounding area for longer amounts of time than those with fewer or without such devices. The effect of the enrichment on the plumage condition remains unclear, since plumage damage developed differently in the groups, independent of their enrichment offer. Hens that were offered fewer devices kept a better plumage than those without or with a high number of devices. Factors other than the enrichment device tested may have influenced the hens’ behavior, but it was well accepted and used by the hens.

**Abstract:**

Feather pecking and cannibalism are prominent problems in modern laying hen husbandry. Among the various approaches to address this issue, environmental enrichment plays a crucial role. In this on-farm study, four winter gardens of an organic farm henhouse were equipped with an automatic enrichment device. Different quantities of downpipes dosing grain on rough-coated pecking plates (PPs) were tested. One group served as a control (CG) without an automatic enrichment device, while the others were offered different numbers of PPs, with one equipped with a doubled amount of PPs (DEG) compared to the other two groups (SEG). Video analyses of the hens’ duration of stay and behaviors in the recorded winter garden area and around the PPs were performed, and regular assessments of the plumage condition were conducted. By the end of the production cycle, no hens with intact plumage were found, with hens in the CG and DEG showing worse scores and earlier deterioration in the plumage condition than in the SEG. The offer of PPs showed a significant influence on the duration of stay in the filmed area. Hens in the DEG stayed significantly longer (mean: 129 s, SD: 126 s) than those in the CG (mean: 79 s, SD: 91 s; *p* < 0.05) and SEG (mean: 75 s, SD: 83 s; *p* < 0.005). On the performed behavioral bouts per hen and minute (CG mean bouts/minute (SD): 5.47 (2.92); SEG mean (SD): 5.33 (2.76); SEG mean (SD): 5.81 (3.24)), no significant influences were detected. Environmental pecking was the behavior most frequently observed in all winter gardens, where, particularly around the PPs, pecking at the device was observed. Therefore, the enrichment device can be assessed as well accepted by the hens in winter gardens. The effect of the device on the plumage condition remains unclear, with external factors probably showing a greater influence than the enrichment.

## 1. Introduction

Red jungle fowl, from which all modern laying hens are descended, spend about 61% of their active daytime ground pecking and approximately 34% thereof ground scratching [1]. Ground pecking and ground scratching are part of foraging behavior, which has special importance for poultry. Even when offered feed ad libitum, laying hens continue to show foraging behavior [2]. Feather pecking and cannibalism are problems that widely occur in modern laying hen husbandry [3]. As their origin, misdirected exploratory and foraging behavior is discussed [4,5]. Plumage damage and injuries occur more often and are more severe in flocks with untrimmed beaks compared to flocks with trimmed beaks [6]. In affected flocks, feather pecking manifests mostly until the 40th week of life (LW), affecting especially the back, tail, and vent region [7,8]. These behavioral problems can result in higher illness and mortality rates [9], lower egg production rates [10], and an increased feed intake [11]. The reasons to develop behavioral disorders are multifactorial, and, therefore, a variety of approaches to address this issue exist [12]. The general goal of those approaches is to optimize husbandry conditions to enable normal behavior and thus increase animal welfare.

For the welfare of laying hens, being able to perform normal behavior is crucial, as is already stated in the “Five Freedoms” [13], and should be possible in the husbandry system. Those systems offering a free-range area are perceived as the most animal-friendly by the consumers [14] and have been on the rise since 2012, when the keeping of laying hens in conventional cages was banned in Germany and EU-wide [15]. In Germany during the year 2022, an estimated 22% of laying hens were housed as conventional free-range hens, and almost 14% as organic hens, both of which are offered a mandatory free-range area in addition to the henhouse [16,17]. Furthermore, an area that is defined as an additional part of the building with outdoor climate conditions and litter [18] must be installed between the henhouse and the free-range area in German laying hen farms [19]. It can be seen as a transitional area from the henhouse to the free-range area, where the hens can acclimatize to the outdoor climate, but without the risk of predation. It offers extra space for the animals to perform natural behavior and for the farmer to offer enrichment materials to encourage such behavior.

Enrichment materials are widely seen as an important part of animal-friendly husbandry systems. Through the offer of enrichment materials, especially material encouraging foraging behavior, the occurrence of behavioral disorders such as feather pecking and cannibalism can be reduced [20,21]. Materials such as pecking stones or alfalfa hay bales are the most frequently provided in Germany [22]. In addition, enrichment materials offered by automated systems are receiving attention. The idea behind most automatic enrichment systems is to offer enrichment to a greater number of animals while at the same time reducing the farmers’ workload. Pipe systems that run through henhouses or winter gardens (WGs) and distribute feed or foraging materials, such as silage, grains, or litter material, have proven their potential to attract large numbers of hens and thus be a step towards increasing animal welfare in laying hen husbandry [23,24].

In this on-farm study, an automatic enrichment system that takes a different approach was evaluated. An enrichment device was installed in the WGs that doses grains on rough-coated plates, triggered by pecks at said plates. Its influence on the number of animals using the WG was studied in a previous study, indicating higher numbers of hens in WGs offering an automatic enrichment device and decreasing interest with a higher age [25]. For the present study, the behavior of the laying hens and duration of stay in the WG were studied under on-farm conditions and the influence of the device on plumage condition was analyzed. The aim of this study was to determine whether the offer of grains over the automatic enrichment device would occupy the hens in the WGs for longer amounts of time and encourage the hens to peck at the rough-coated plates. Furthermore, an effect of the device on plumage condition and thus, consequently, feather pecking was to be investigated.

## 2. Materials and Methods

### 2.1. Animals and Housing

Data collection took place on an organic laying hen farm in Germany keeping hens in accordance with EU regulations on organic production [17] and the standards of the organic association Naturland [26]. A total of 11,993 laying hens (Lohmann Brown Lite, Lohmann Tierzucht GmbH, Cuxhaven, Germany), split into four flocks (of about 3000 hens each), were housed in four separate units in the same henhouse.

Each group had a separate compartment with an aviary (Natura Step, Big Dutchman International GmbH, Vechta-Calveslage, Germany) in the henhouse and daily access to a winter garden (WG) and a free-range area. Feed, water, and nests were provided in the henhouse only, while enrichment materials were offered in the WG. In both areas, the litter consisted of straw pellets. Each WG covered an area of 160 m^2^ (3.20 m × 50.00 m) and had to be crossed to get from indoors to the free-range area. Two WGs (3 and 4) were oriented to the east, the other two (1 and 2) to the west. They were accessible during the day (at a minimum from 09:00 to 20:00). Each WG had 10 openings of 2 m width that enabled the hens to enter the henhouse and 10 openings of 2–4 m width that led to the free-range area. In this area, each hen had 4 m^2^ of grassland with vegetation offering shade and protection available. The outlets outside into the free-range area were opened at the latest at 10:00 and during the first observational period were closed for some days due to bad weather to protect the animals’ health.

The flocks were visited for weighing and plumage scoring 10 times during production: LW 19, 24, 28, 31, 35, 41, 54, 62, 70, and 77. On these occasions, climate measurements inside the henhouse were also taken (temperature and humidity: testo 174 T, Testo SE & Co. KGaA, Titisee-Neustadt, Germany; NH_3_: Dräger X-am 5100, Drägerwerk AG & Co. KGaA, Lübeck, Germany). Transport to the slaughterhouse took place at LW 78.

**Figure 1:**
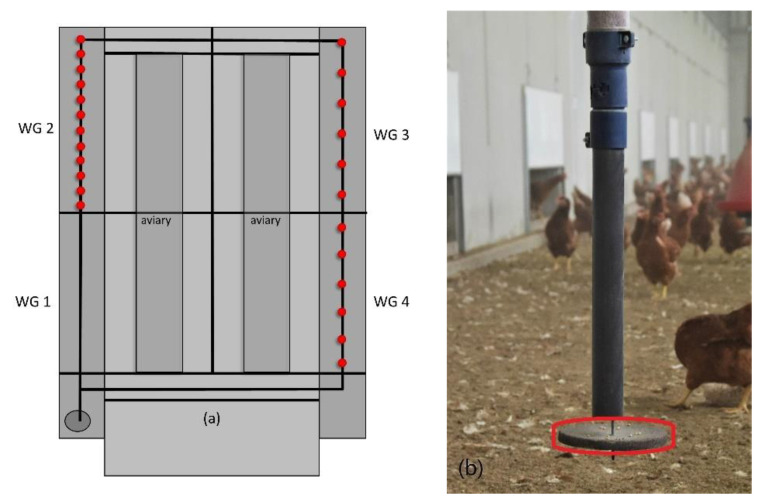
(**a**) Scheme of the henhouse and winter gardens (WGs 1–4) with the downpipes with pecking plates indicated with red dots; (**b**) photograph of the enrichment device with the pecking plate circled in red (photo: A. Riedel). Adapted from [25].

An automatic enrichment device (PickPuck, Big Dutchman International GmbH, Vechta-Calveslage, Germany) was installed in the WGs before the hens were first allowed access (Figure 1a). It included a 6 m^3^ capacity silo that was located beside WG 1. A pipe system was installed under the ceiling of WGs 1–4, which originated from the silo and inside; a chain with driving plates transported material from the silo through the henhouse. In WGs 3 and 4, six downpipes were attached approximately every 7.2 m, following the manufacturer’s recommendations of one downpipe for 500 hens. These two WGs are summarized as the “single enriched group” (SEG). In WG 2, the number was doubled to 12 downpipes in a distance of approx. 3.6 m from one to another. This means one downpipe for 250 hens and was described as the “double enriched group” (DEG). Each downpipe ended in a dosing mechanism that could be adjusted in three steps, from which a rough-coated pecking plate (PP) hung below. During the study, the middle step for pellets or larger grains was used. The plate swung freely on a pendulum that influenced the material dosage (Figure 1b). The hens could trigger the dosage by pecking at the plate. In WG 1, used as a control group (CG), no downpipes or pecking plates (PPs) were installed. During this study, wheat grains were used to fill the downpipes and run the enrichment device. Since the dosage of grains was triggered by the hens, they could access the material ad libitum. As a base enrichment, pecking blocks, hay/straw, and alfalfa hay bales hanging in nets were offered to the hens in all groups. Additionally, each group had access to two boxes offering material for dust bathing inside the henhouse.

### 2.2. Data Collection and Behavioral Observation

Production data as well as feed and water consumption were not recorded separately for the four groups but for the entire henhouse.

During the 10 on-farm visits, 30 hens in the CG, DEG, and SEG were randomly selected inside the henhouse. Every hen was weighed (manual poultry scales BAT1, VEIT Electronics, s.r.o. Moravany, the Czech Republic) and checked for feather loss and skin lesions following the modified protocols of Giersberg et al. [27] (Table 1). Scores were given for the five body regions, the neck, back, tail, wing, and belly, using a six- and four-point scheme. Thereafter, the upper beak length of every hen was determined to evaluate a potential abrasive effect of the pecking plates using a measuring tape with 1 mm accuracy (Prym Maßband Junior 150 cm, Prym Consumer Europe GmbH, Stolberg, Germany). The measurement was taken from the inner angle of the right nostril to the tip of the beak.

In each WG, one camera (EverFocus EQ900F eZ. HD, EverFocus Co., New Taipei City, Taiwan) was installed and connected to receivers (Monacor AXR-108 8CH Hybrid, MONACOR INTERNATIONAL GmbH & Co. KG, Bremen, Germany). The filmed area included a section measuring 3.20 m × 6.00 m (19.2 m^2^) with one PP in the DEG (WG 2) and SEG (WG 3). In WG 4, no recordings of the PPs were made due to a slightly shifted camera angle caused by the separation compartment located here.

Recordings from the peak of the laying period (LW 31/32, Phase A), the middle of the laying period (LW 50/51, Phase B), and the end of the laying period (LW 64/65/71, Phase C) were analyzed. Therefore, on two days of each phase in the time between 14:00 and 16:00, 10 randomly selected hens were observed. This time was chosen on the basis of hen counts from the same WGs [25], indicating times when the area was well frequented and no feeding times in the henhouse were programmed. The program GoldenRatio (Version 3.1, Markus Welz, Krailling, Germany) was used to outline the observed area consistently in every video.

Behavioral observations and measurements of the duration of time the hens spent in the area were performed in all four groups on 20 hens per phase and WG (240 hens in total). The selection of focus animals was made by choosing the first hen entering the area through the outlet from the henhouse at 10 min intervals. For each hen, the time of entering and leaving the observational area, read on the internal timer of the video, was noted as well as the direction in which the hen left the area (towards the henhouse, the free-range, or the rest of the WG). In addition, the behaviors performed were noted, following a previously developed ethogram (Table 2). Bouts of the different behaviors were counted, a behavior being counted as one bout as long as it was continuously performed. Each bout ended when a hen stopped the behavior for more than 4 s, performed another behavior, or left the area. In addition, pecking behavior was observed, defining repeated pecks at the same conspecific or object as one bout, counting it as one behavior, based on a study by Rieke et al. [28]. All behavioral scans were conducted by one trained observer.

For observations of the behaviors at the enrichment device, in the area close to the PP (within one hen’s body length), 20 hens per phase were additionally observed during the same time of day in the SEG and DEG, resulting in a total of 120 observations. The observation of the PP use was performed according to the same premises as the WG use, starting when a hen pecked at the plate and ending with the animal leaving the area close to the PP.

### 2.3. Statistical Analysis and Data Processing

The data were stored and organized in Microsoft Excel 2016 (Microsoft Corporation, One Microsoft Way, Redmond, WA, USA), which was also used to create graphs on plumage scores. Statistical analyses were performed and the remaining graphs created using RStudio Version 1.4.1717 (Integrated Development for R. RStudio, PBC, Boston, MA, USA [29]).

An ANOVA was performed to determine group differences in the hens’ weight for every LW when the hens were weighed and also for the beak length. For the comparability of plumage scores, the worst score achieved in one of the five body regions was taken for each hen and occurrences were compared descriptively. Pairwise comparisons of the maximum scores per hen were performed within the groups and the LW using Kruskal–Wallis tests and the post hoc Dunn test with the Holm correction. The data on skin lesions were analyzed in the same way.

For the behavioral observations in the WGs, the observations from WG 3 and WG 4 (with same experimental conditions regarding the PPs) were summarized as the SEG and the mean results were used for evaluation. For better comparability, all observations were calculated as bouts/hen/minute. Due to the lack of a normal distribution, the correlation was calculated using the Spearman’s correlation. Kruskal–Wallis tests were performed to find significant differences between studied influencing factors on the hens’ behavior. Afterwards, pairwise Wilcoxon rank tests using a Bonferroni correction were used to calculate individual group differences. For tests comparing only two groups, such as in the usage of the PPs (only WG2 and WG3) or the comparison of behaviors in the WG and the PP, Mann–Whitney U tests were performed.

## 3. Results

### 3.1. Climate and Production Data

Climate measurements inside the henhouse in the CG, DEG, and SEG showed that the hens’ environmental conditions stayed in the range of the recommended parameters and did not differ between groups. Temperatures were lowest during winter months, with the lowest values of 10 °C, and the highest in summer with up to 30 °C inside the henhouse.

The average feed and water consumption as well as the laying performance of the flock were within the tolerance range of the breeder’s recommendations (Table 3). The laying rate peaked in LW 28 with 95.1% and stayed above the breeder’s recommendation for most of the time.

**Figure 2:**
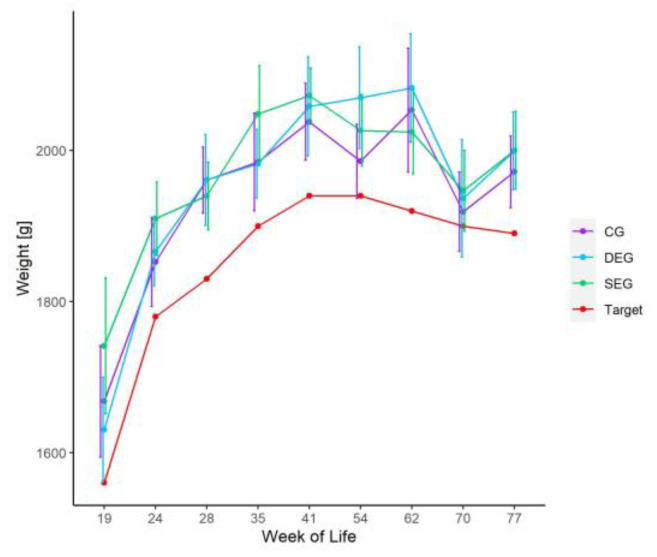
Mean live weights and standard deviation of the hens (Lohmann Brown Lite, Lohmann Tierzucht GmbH, Cuxhaven, Germany) in the three groups (CG = control group, SEG= single enrichment group, DEG= double enrichment group), determined via manual weighing of 30 hens per group and the target weights as recommended by the breeder [30] during the husbandry period (weeks of life 19–77).

The mean live weights did not differ significantly between the groups at any of the evaluated days (Figure 2) and exceeded the recommendations provided by the breeder [30].

### 3.2. Plumage Condition

**Figure 3:**
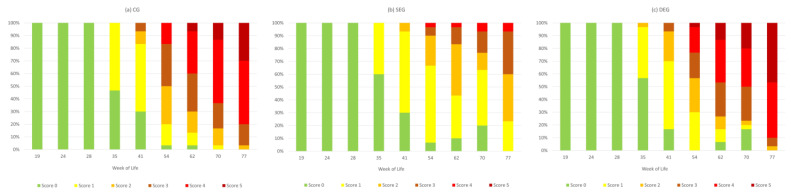
Percentage of animals with a plumage score of 0 (intact plumage) to 5 (<75% of feathers missing) in at least one body region in the three groups: (**a**) CG = control group, (**b**) SEG = single enrichment group, and (**c**) DEG = double enrichment group during the husbandry period (weeks of life 19–77).

A comparison of the plumage condition during the husbandry period for the three groups is depicted in Figure 3. While no plumage damage was evident in any of the groups during the first three visits, this changed in LW 35. At this visit, 53% of the scored hens in the CG showed a feather score of 1 in at least one body region. In the DEG and SEG, score 1 was assigned to 40% of the hens. From this day onwards, the proportion of body regions with a feather score of 0 decreased steadily, while higher scores were assigned more frequently. Exceptions were the DEG and SEG in LWs 62 and 70, when the proportion of hens with score 0 increased (from 0% (LW 54) to 7% (LW 62) and then 17% (LW 70) in the DEG and from 7% (LW 54) to 10% (LW 62) and 20% (LW 70) in the SEG). At the end of the husbandry period, no hens with intact plumage remained. Plumage score 4 was first assigned in LW 54, where in CG 17%, in DEG 20 %, and in SEG 3% of the scored hens were affected. While in the SEG the proportion of hens with scores 4 and 5 remained low (a maximum of 7%), this made up a larger proportion in the CG (40% in LW 62, 63% in LW 70, and 80% in LW 77) and the DEG (46% in LW 62, 50% in LW 70, and 90% in LW 77). Score 5 was never assigned to a hen in the SEG. The statistical evaluation showed a significantly worse plumage condition in the CG and DEG than in the SEG (*p* < 0.001). It can also be seen that, as of LW 41, the plumage condition was scored significantly worse than in the previous LW (*p* < 0.05), with the exception of LW 62 to LW 70 when no significant differences occurred.

**Figure 4:**
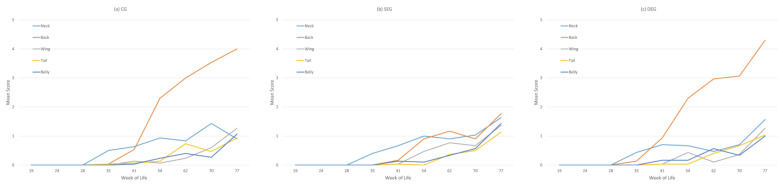
Mean plumage score of 30 hens/group/week of life on a scale of 0 (intact plumage) to 5 (<75% of feathers missing) in five assessed body regions (neck, back, wing, tail, and belly) in the three groups: (**a**) CG = control group, (**b**) SEG = single enrichment group, and (**c**) DEG = double enrichment group during the husbandry period (weeks of life 19–77).

In all three groups, the first feather losses (after LW 28) occurred mostly in the neck region, while in LW 41 more regions were affected and, by LW 54, the mean score of the back exceeded that of the neck (Figure 4). In LW 35, 40% (SEG) to 50% (CG) of the hens showed single missing feathers on the neck resulting in a mean score of 0.44 there. In LW 41, the mean scores on the neck increased to a mean of 0.66 with only slight differences between the groups. At the same time, a higher mean score was observed on the back (mean: 0.54), with hens in the DEG reaching higher mean scores there than in the neck. In the SEG, in contrast, the mean score for the back region was 0.17. All other body regions showed lower mean scores in all groups, these irregularly increasing during the ongoing production period resulting from the proportion of body regions with a feather score of 0 decreasing consistently, while higher scores were assigned more frequently. While the mean score on the back in the CG (4) and DEG (4.3) exceeded those of the other body regions (CG: 0.9–1.27; DEG: 1–1.56), it stayed similar in the SEG (lowest: tail, 1.13; highest: back, 1.77). This highlights the importance of the back region.

### 3.3. Skin Lesions

Skin lesions did not occur in any scored hen up to LW 62. In that LW, 13% of the hens in the CG and 7% in the DEG showed a lesion score of 1. During the following visit in LW 70, the occurrence of injuries declined to 10% in the CG and 3% in the DEG, while, in LW 77, 13% of the hens in the CG, 17% in the DEG, and, for the first time, 3% of hens in the SEG showed a lesion score of 1. None of the assessed hens showed a skin lesion of score 2 or 3. During the entire husbandry period, the hens in the CG showed significantly more skin lesions than those in the SEG (*p* < 0.05).

### 3.4. Beak Measurements

The beak length did not differ significantly between the groups (Table 4) and was distributed around a mean of 19.3 mm (SD: 0.55 mm). During the entire husbandry period, the beaks tended to become shorter (mean beak length in LW 19: 20.1 mm; mean beak length in LW 77: 19.1 mm).

### 3.5. Behavioral Observations in the WG

**Figure 5:**
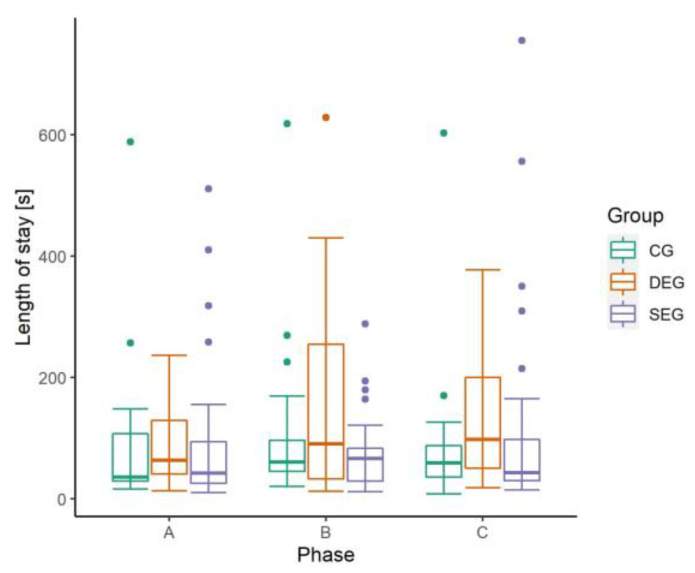
Boxplots with dots indicating individual outliners of the length of stay(s) of laying hens in the observed area in the winter garden during different phases (Phase A: peak of laying period, weeks of life (LW) 31/32; Phase B: middle of laying period, LWs 50/51; Phase C: end of laying period, LWs 64/65/71) and in three groups (CG = control group, SEG = single enrichment group, DEG = double enrichment group).

The average stay in the observed area of the WG lasted about 90 *s* (SD: 100 s, min: 8 s, max: 10 min and 28 s). The length of stay varied between the groups (Figure 5). Hens in the DEG stayed significantly longer (mean: 129 s, SD: 126 s) in the WG than hens offered no automatic enrichment device (CG; mean: 79 s, SD: 91 s; *p* < 0.05) and hens offered the standard amount of the enrichment device (SEG; mean: 75 s, SD: 83 s; *p* < 0.005). The production phase did not show a significant influence on the length of stay, but a trend towards a shorter stay in Phase A (mean: 81 s, SD 89 s) than Phase B (mean: 96 s, SD: 109 s) and C (mean: 92 s, SD 101 s) was observable.

Most hens left the observed WG area after 30–60 s. Independent of the length of stay, most animals stayed in the WG, only leaving the observed area (68%). A total of 18% of the hens left the WG to go to the free-range area, while 14% returned inside the henhouse after their stay there. Hens leaving the WG to go to the free-range area tended to stay for a shorter period of time (mean: 70 s, SD: 79 s) than hens that left to go to the henhouse (mean: 97 s, SD: 98 s) or the rest of the WG (mean: 93 s, SD 79 s).

The duration of stay in the WG and the sum of observed behaviors correlated significantly (*p* < 0.005), where during a longer stay significantly more behaviors were performed. Contrary to this, the amount of bouts/hen/min decreased with longer stays. No significant influence of the group on the sum of performed behaviors was found (Table 5). In contrast to this, it was shown that the production phase had a significant influence on the overall number of performed behaviors. During the end of production, significantly fewer behaviors were observed per hen and minute than in the two previous phases (Phase A: *p* < 0.005; Phase B: *p* < 0.05).

On average, 4.9 bouts of EP per hen and minute (SD: 2.9 bouts/hen/min) were displayed, making it the most frequently displayed behavior with a mean of 86% of all observed behaviors. The number of offered pecking plates (PPs) in the WG influenced the mean percentage only slightly (CG: 89%, SEG: 86%, DEG: 83%). The offer of PPs did not influence the number of observed bouts of EP significantly, while the production phase showed a significant decrease in EP (*p* < 0.005) during the end of production compared to the other two phases. Pecking at the pecking plate (PPP) was observed in about 6% of hens in general and most frequently in Phase A (*p* < 0.05). The number of offered PPs did not influence the amount of bouts/hen/min towards the PP. Feather pecking (FP) was rarely observed in the CG and the SEG, but, in the DEG, significantly more hens showed FP than in the SEG (*p* < 0.001). This can be traced back to very few hens in the DEG showing several FP bouts (DEG max: 6.75 bouts/hen/min) compared to sporadically observed single bouts in the other groups (CG max: 1.2 bouts/hen/min; SEG max: 0.4 bouts/hen/min). Comfort behavior, dust bathing, and scratching were rarely displayed and showed no significant differences in between phases or groups.

Hens that left the WG to go to the free-range area showed less EP (mean: 3.8 bouts/hen/min, SD: 2.6 bouts/hen/min) than hens that left to go to the henhouse (mean: 5.3 bouts/hen/min, SD: 3.2 bouts/hen/min) and the rest of the WG (mean: 5.1 bouts/hen/min, SD: 2.9 bouts/hen/min, *p* < 0.05). Other behaviors were not significantly influenced by the direction in which the hens left.

### 3.6. Behavior around the Enrichment Devices

In general, animal behavior differed between the observed areas. The observed hens stayed around the PPs for between 8 s and 3 min 43 s (mean: 49 s, SD: 45 s). The amount of offered enrichment devices influenced the duration of stay at the PP significantly (*p* < 0.05). Hens in the SEG occupied themselves in the area around the PP for a mean of 55 s (SD: 43 s), while hens in DEG stayed for a mean of 44 s (SD: 47 s). The production phase of the observations did not show a significant influence on the duration of stay near the PP.

The sum of expressed behaviors within the PP area differed significantly between the numbers of offered PPs (*p* < 0.01) but not between the phases (Table 6). Around the PP, the hens mostly showed pecking behavior towards the pecking plate (mean: 49%), which was displayed by 92% of the observed hens, while environmental pecking was the second most common behavior (mean: 45%). While EP was observed significantly more in the DEG (*p* < 0.001), PPP occurred more often in the SEG (*p* < 0.05). The production phase also influenced the display of EP and PPP in a completely different way, resulting in significantly more EP during Phase B than Phase C (*p* < 0.01) and significantly less PPP in Phase B than in Phase A and Phase C (both *p* < 0.001). Scratching behavior was observed significantly more often in the SEG than in the DEG (*p* < 0.05) and less in Phase A than in Phase C (*p* < 0.05). The occurrence of comfort behavior and dust bathing did not differ significantly between the groups or phases. Feather pecking was not observed around the PP.

## 4. Discussion

The present on-farm study aimed to explore the influence of an automatic enrichment device dosing grain via PPs on laying hen behavior in the WG and FP occurrence. For this purpose, video recordings were used for behavioral observations, and the hen’s plumage and integument were scored regularly during the production process. Animal behavior was compared within three phases and three groups with different quantities of PPs. The groups differed in the severity of their plumage damage and in their behavior, primarily the length of stay in the WG and around the PPs. The production phase showed no significant effects on the behavior, with the exception of pecking at the PP, but with later weeks of life the hens’ plumage condition deteriorated. The high proportions of pecking at the PP indicated the attractiveness of the enrichment device for the hens.

At ten points in the production phase, a sample of 90 hens (divided into three groups) was examined for weight, plumage damage, and beak length. The evaluation showed a steady and significant deterioration in the plumage condition with increasing age. While skin lesions were only found sporadically in single hens at later stages of production, missing feathers were found at an earlier stage. Later, featherless areas, especially on the back of the hens, were detected. This matches findings by Bilcik et al., (1999) as well as Bright (2009), who found worst plumage scores and earliest feather loss in the back, rump, tail, and belly region [7,8]. In contrast to their findings, the feather loss in the flock in the present study started at the neck and continued at the back (including rump) and tail. A probable reason could be heat-stress-induced neck molt, as a partial molt during the summer, which would explain the early feather loss. Furthermore, an interim improvement in plumage condition in the neck region could be observed, indicating the regrowth of feathers. The groups CG (without an enrichment device) and DEG (with double the enrichment devices) showed significantly more hens with worse plumage scores than the SEG (with a single enrichment device). To evaluate the effect of the device on the plumage damage in more detail, further experiments in a more controlled environment are necessary. Since both the groups without and with double the enrichment devices were equally affected by FP, other effects seem to have had a significant influence on FP. The offer of enrichment materials by the automatic device alone therefore did not help to influence the plumage damage. Apparently, other effects, such as the location of the individual groups in the henhouse, the WGs facing towards the sun, and the wind direction, or other not investigated effects, also influenced behavior besides the PPs. Both the WGs of the CG and DEG were located on the western side of the henhouse, but shared no other similarities that distinguished them from the SEG where the hens had better plumage condition scores. Manifold factors can have an influence on FP and therefore plumage condition, such as feed form [10], enrichment offer [31,32], disease [33], or free-range use [33,34,35]. The feed form and enrichment offer were identical on both sides of the henhouse, which makes these factors seem unlikely to have differently influenced the plumage condition in this study. Another limitation consisted of the lack of replicates for the group without and with the double amount of enrichment material. Due to the nature of the data collection as an on-farm study, the setting did not allow replicates. The influence of the position of the WG around the henhouse and the enrichment device can therefore not be separated conclusively. Furthermore, inaccurate results could arise due to the relatively small sample size, which makes it hard to compare the different groups. Nevertheless, such sample sizes are common in on-farm studies and are widely seen as a good indicator for the occurrence of FP and cannibalism [24,34,36]. Even if the captured hens were randomly selected from all henhouse areas, it is still questionable whether they formed a representative sample of the actual population. Since no results on individual hens’ use of the PP exist, it is also doubtful if the hens scored regarding plumage damage were ones that also benefited from the enrichment.

The same applies to the measurements of the beak, which fluctuated between single measurements but did not differ between the groups. Furthermore, no correlation of the hens’ weight and the length of their beak was found. Again, the measurements were taken on randomly caught hens, with no information regarding the individual use of the enrichment device. However, this could also result from the relatively inaccurate measurement method using a tape measure with 1 mm accuracy. More accurate measuring methods and tools exist, for example, caliper measurements [37] or beak photographic evaluation [38]. Repeated measurements on the same hens could be a more appropriate means to evaluate the beak length over the production phase.

The evaluation of the results on animal behavior is complicated by the fact that the study conducted here was an on-farm experiment with a variety of external factors that could modulate animal behavior. The existing offer of various enrichment materials in addition to the PPs could therefore have been enough to motivate the hens to enter the WG and forage. The behavior mostly observed in this study was environmental pecking, making up between 83% and 89% of all observed behaviors in the WG and 29–67% around the PP. Environmental pecking is generally seen as one of the most often performed behaviors. Dawkins observed it in red jungle fowl during 60% of all minutes of observation [1]. The documented high percentage in the present study could have been caused on the one hand by the observation of just the WG area, where this behavior is promoted actively by the offer of enrichment materials and litter. On the other hand, an ethogram was used that focused on foraging and comfort behaviors and did not include behaviors such as walking and standing. The expression of individual behaviors (excluding pecking at conspecifics) did not differ significantly between the three groups. This could indicate that, in general, all hens, independent of the offer of enrichment materials, performed natural behaviors in the area equally. An effect of the enrichment device on behavior could therefore be better observed in a more barren environment. While over the different phases almost all behaviors were observed similarly frequently, the number of environmental pecks per hen per minute decreased significantly during the last phase.

The results showed that in the DEG the animals stayed significantly longer within the observed area than in the other groups, indicating it was most attractive for longer stays. If this results from the offer of the doubled amount of PPs or other factors remains open. A periodic offer of silage as enrichment in the WG was proven to extend the length of stay in the area in comparison to an unenriched WG in a study of Giersberg et al. [23]. In their study, the hens stayed in the unenriched WG for shorter amounts of time, indicating that in this study an effect of the enrichment offered in all WGs was notable. Furthermore, the duration of time spent in the WG seems to be influenced by flock size [39] and daytime [40], which complicates comparisons between different studies. The light conditions in the WGs may have had an influence on the length of stay and behavior. Since the WGs from the SEG were oriented in the eastern direction, direct sunlight shone mostly in the morning hours, while the CG and DEG had sunshine in the afternoon, when the observations were made. Further behavioral observations from the late morning could help to reduce the probable effects of the daytime and lighting conditions.

The behavior of pecking at conspecifics (FP) was observed in the DEG significantly more often than in the SEG, corresponding with the findings of the plumage scoring, which showed worse damages in the DEG. This finding, however, leaves out the CG with bad plumage scores but almost no observed FP. Since no differentiation between aggressive pecking, severe FP, and gentle FP was made during the observation, a direct correlation between the observation of FP and the occurrence of plumage damage cannot be made. It could be that the number of focus animals was not high enough to capture this behavior in all WGs, or the other groups did not show feather pecking in the WG area. Furthermore, Kjaer found out that feather pecking rates rose over the day and were highest 14 h after the lights came on [41], which would be the evening hours in this study, when no video recordings were evaluated.

Those hens leaving the WG to go to the free-range area stayed there for a shorter period of time and showed less environmental pecking than conspecifics that left to go to the henhouse or stayed in the WG. In general, laying hens were found to spend more time in the free-range area than the WG [40], with hens from smaller flocks staying in the WG longer than conspecifics in larger groups [39]. Moreover, higher percentages of hens were observed to use the WG than the free-range area [39]. In addition, Larsen et al., (2017) observed that foraging is the behavior mostly performed in the free-range area, which is affected by location and time of day [42]. Therefore, it can be assumed that the WG is used for pecking behavior mainly by the hens that do not enter the free-range area.

Around the pecking plate, most animals were interested in the device and showed high counts of pecking behavior directed towards the device, with hens in the SEG pecking at the PP significantly more often than in the DEG, while, in the DEG, significantly more EP was recorded around the PP. This could originate in the higher offer of PPs in the DEG, allowing the hens to perform PPP on more PPs of which only one was observed. With 49% of all pecks directed towards the PP and 45% towards other environmental structures, the enrichment device was an attractive object to peck at for hens in close proximity to the PP. In the middle of the laying period, significantly less PPP was observed, while simultaneously the EP increased. A temporary decrease in interest in the PP could originate from habituation to the device, making it less interesting after a while, with hens directing pecks for foraging and exploring to other surroundings. Another explanation could be the sometimes-unpredictable functionality of the device. It could occur that single broad beans, which contaminated the wheat grains, clogged the dosing mechanism for a certain time, so that pecks at the PP no longer dispensed grains and the hens lost interest in the device. Other times, when the downpipes were freshly filled with material, their interest could rise due to the probably more successful pecks. Since not every peck resulted in the same result, the device was more rewarding to some hens than to others; other hens probably focused on pecking grains that fell from the PP into the litter, which would count as environmental pecking if observed.

## 5. Conclusions

In conclusion, the results of this on-farm study showed the acceptance of and behavior around an automatic enrichment device. Up to 6% of all the hens in the WG area made use of the PP simultaneously and hens occupying themselves with the device directed the greatest proportion of their behaviors towards it. Nonetheless, an influence of the device on plumage damage cannot be evaluated conclusively due to the lack of replicates of the observed groups and their diverging conditions. Whether such an enrichment device is beneficial for animal welfare and therefore should be used on commercial farms depends on many factors and could not be assessed finally in the present study.

## Figures and Tables

**Table 1:** Scoring system for the plumage evaluation and skin lesion evaluation used for the five body regions, the neck, back, tail, wing, and belly ([27], modified).

Score	Feather Loss	Injuries
0	no feather loss, intact plumage	no visible skin lesion
1	single feathers missing	maximum of 2 small lesions (<1 cm)
2	<25% of feathers missing	3 or more small lesions (<1 cm) or at least 1 large lesion (>1 cm)
3	>25% to <50% of feathers missing	massive lesions (>3 cm)
4	>50% to <75% of feathers missing	-
5	>75% of feathers missing	-

**Table 2:** Ethogram of continuous observations of general behavior in the winter garden and around the automatic enrichment device ([28], modified).

Behavior	Description
Environmental pecking (EP)	Pecks directed at any surface, including ground pecking and object pecking (excluding pecks at the enrichment device)
Pecking at conspecifics (FP)	Careful, aggressive, or strong pecks towards a conspecific’s head or plumage
Pecking at pecking plate (PPP)	Pecks directed at the rough-coated plate or downpipe of the enrichment device
Comfort behavior (Comf)	Includes preening, body shakes, wing flaps, leg and wing stretches, and tail wags
Dust bathing (DB)	Manipulation of substrate with the wings, feet, tail, and/or beak while lying in the litter with some or all feathers fluffed
Scratching (Scra)	Standing and scratching repeatedly the litter with one foot or two feet in a backward movement

**Table 3:** Production data (weeks of life 22–78) of the project flock and breeder’s recommendations [30].

	Project Flock	Recommendation
Mean laying rate (%)	89.6	88.2
Total hen-housed eggs	351	354 (to 80 weeks)
Mean food consumption/hen/day (g)	125.5	113–123
Mean water consumption/hen/day (ml)	206.5	210 (at 21 °C)
Livability (%)	90.3	90–92 (to 90 weeks)

**Table 4:** Mean beak length, standard deviation, and minimum and maximum values in mm measured at nine visits in weeks of life 19, 24, 28, 31, 35, 41, 54, 62, 70, and 77, in three groups (CG = control group, SEG= single enrichment group, DEG = double enrichment group).

Group	Mean (SD)	Min	Max
CG	19.39 (0.59)	18.37	20.23
SEG	19.27 (0.55)	18.47	20.08
DEG	19.34 (0.58)	18.53	20.13

**Table 5:** Mean and standard deviation (SD) of the different behaviors per minute and hen observed in the WG of three groups (CG = control group, SEG = single enrichment group, DEG = double enrichment group) and different phases during production (Phase A: peak of laying period, weeks of life (LWs) 31/32; Phase B: middle of laying period, LWs 50/51; Phase C: end of laying period, LWs 64/65/71).

Behavior in the WG	Mean (SD) Bouts/Hen/Min	CG	SEG	DEG	Phase A	Phase B	Phase C	All
Environmental pecking (EP)		5.06 (2.91) ^a^	4.72 (2.82) ^a^	4.98 (3.01) ^a^	5.3 (2.76) ^a^	5.44 (3.12) ^a^	3.86 (2.49) ^b^	4.87 (2.88)
Pecking at conspecifics (FP)		0.04 (0.2) ^ab^	0 (0.04) ^b^	0.27 (1.02) ^a^	0.18 (0.88) ^z^	0.02 (0.11) ^z^	0.05 (0.26) ^z^	0.08 (0.54)
Pecking at pecking plate (PPP)		/	0.12 (0.67) ^a^	0.14 (0.48) ^a^	0.22 (0.83) ^z^	0.01 (0.11) ^y^	0.06 (0.36) ^zy^	0.1 (0.54)
Comfort behavior (Comf)		0.38 (1.17) ^a^	0.21 (0.61) ^a^	0.15 (0.33) ^a^	0.12 (0.38) ^z^	0.18 (0.44) ^z^	0.41 (1.13) ^z^	0.23 (0.74)
Dust bathing (DB)		0.01 (0.04) ^a^	0 (0.03) ^a^	0.01 (0.08) ^a^	0.01 (0.06) ^z^	0.01 (0.06) ^z^	0 (0.02) ^z^	0.01 (0.05)
Scratching (Scra)		0.18 (0.49) ^a^	0.36 (1.03) ^a^	0.25 (0.62) ^a^	0.33 (0.98) ^z^	0.26 (0.71) ^z^	0.28 (0.78) ^z^	0.29 (0.83)
Sum of observed behaviors (All)		5.47 (2.92) ^a^	5.33 (2.76) ^a^	5.81 (3.24) ^a^	6.08 (2.77) ^z^	5.84 (3.14) ^z^	4.54 (2.62) ^y^	5.49 (2.92)

Statistical significances between groups and phases indicated using different letters (*p* < 0.05).

**Table 6:** Mean and standard deviation (SD) of the different behaviors observed per minute and hen around the enrichment device in two groups (SEG = single enrichment group, DEG = double enrichment group) and different phases during production (Phase A: peak of laying period, weeks of life (LWs) 31/32; Phase B: middle of laying period, LWs 50/51; Phase C: end of laying period, LWs 64/65/71).

Behavior around the PP	Mean (SD) Bouts/Hen/Min	SEG	DEG	Phase A	Phase B	Phase C	All
Environmental pecking (EP)		2.51 (2.48) ^a^	5.44 (4.6) ^b^	3.24 (2.7) ^zy^	6.17 (5.27) ^z^	2.5 (2.26) ^y^	3.98 (3.96)
Pecking at conspecifics (FP)		0 (0)	0 (0)	0 (0)	0 (0)	0 (0)	0 (0)
Pecking at pecking plate (PPP)		3.93 (2.57) ^a^	3.21 (3.67) ^b^	4.59 (2.98) ^z^	1.76 (2.32) ^y^	4.38 (3.38) ^z^	3.57 (317)
Comfort behavior (Comf)		0.02 (0.11) ^a^	0.06 (0.44) ^a^	0 (0) ^z^	0.02 (0.14) ^z^	0.09 (0.55) ^z^	0.04 (0.32)
Dust bathing (DB)		0 (0) ^a^	0.01 (0.05) ^a^	0 (0) ^z^	0 (0) ^z^	0.01 (0.06) ^z^	0.003 (0.03)
Scratching (Scra)		0.3 (0.88) ^a^	0.13 (0.7) ^b^	0.04 (0.25) ^z^	0.1 (0.48) ^zy^	0.52 (1.24) ^y^	0.22 (0.79)
Sum of all observed behaviors		6.76 (2.57) ^a^	8.85 (4.14) ^b^	7.86 (3.18) ^z^	8.05 (4.14) ^z^	7.49 (3.45) ^z^	7.81 (3.58)

Statistical significances between groups and phases indicated using different letters (*p* < 0.05).

## Data Availability

The data presented in this study are available on request from the corresponding authors. The data are not publicly available due to privacy concerns.
